# 5-Bromo-2-methyl­pyridine *N*-oxide

**DOI:** 10.1107/S1600536808013391

**Published:** 2008-05-10

**Authors:** Bo-Nian Liu, Shi-Gui Tang, Hao-Yuan Li, Ye-Ming Xu, Cheng Guo

**Affiliations:** a College of Science, Nanjing University of Technolgy, Xinmofan Road No. 5, Nanjing 210009, People’s Republic of China; b College of Life Science and Pharmaceutical Engineering, Nanjing University of Technology, Nanjing 210009, People’s Republic of China

## Abstract

In the mol­ecule of the title compound, C_6_H_6_BrNO, the methyl C and oxide O atoms lie in the pyridine ring plane, while the Br atom is displaced by 0.103 (3) Å. In the crystal structure, inter­molecular C—H⋯O hydrogen bonds link the mol­ecules into centrosymmetric dimers.

## Related literature

For related literature, see: Ochiai (1953 ▶). 
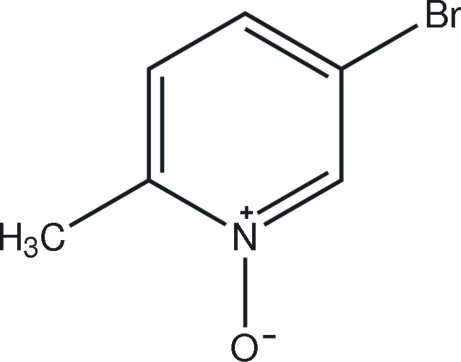


## Experimental

### 

#### Crystal data

C_6_H_6_BrNO
*M* *_r_* = 188.03
Monoclinic, 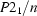

*a* = 7.3060 (15) Å
*b* = 11.351 (2) Å
*c* = 8.4950 (17) Å
β = 111.01 (3)°
*V* = 657.7 (3) Å^3^
*Z* = 4
Mo *K*α radiation
μ = 6.16 mm^−1^
*T* = 294 (2) K
0.10 × 0.05 × 0.05 mm

#### Data collection

Enraf–Nonius CAD-4 diffractometer
Absorption correction: ψ scan (North *et al.*, 1968 ▶) *T*_min_ = 0.578, *T*_max_ = 0.748
1275 measured reflections
1180 independent reflections
747 reflections with *I* > 2σ(*I*)
*R*_int_ = 0.036
3 standard reflections frequency: 120 min intensity decay: none

#### Refinement

*R*[*F* ^2^ > 2σ(*F* ^2^)] = 0.063
*wR*(*F* ^2^) = 0.162
*S* = 1.05
1180 reflections
76 parameters
H-atom parameters constrained
Δρ_max_ = 0.87 e Å^−3^
Δρ_min_ = −0.69 e Å^−3^

### 

Data collection: *CAD-4 Software* (Enraf–Nonius, 1989 ▶); cell refinement: *CAD-4 Software*; data reduction: *XCAD4* (Harms & Wocadlo, 1995 ▶); program(s) used to solve structure: *SHELXS97* (Sheldrick, 2008 ▶); program(s) used to refine structure: *SHELXL97* (Sheldrick, 2008 ▶); molecular graphics: *PLATON* (Spek, 2003 ▶); software used to prepare material for publication: *SHELXTL* (Sheldrick, 2008 ▶).

## Supplementary Material

Crystal structure: contains datablocks global, I. DOI: 10.1107/S1600536808013391/hk2459sup1.cif

Structure factors: contains datablocks I. DOI: 10.1107/S1600536808013391/hk2459Isup2.hkl

Additional supplementary materials:  crystallographic information; 3D view; checkCIF report

## Figures and Tables

**Table 1:** Hydrogen-bond geometry (Å, °)

*D*—H⋯*A*	*D*—H	H⋯*A*	*D*⋯*A*	*D*—H⋯*A*
C1—H1*A*⋯O^i^	0.93	2.41	3.264 (11)	153

Symmetry code: (i) 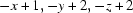.

**Fig. 1.**
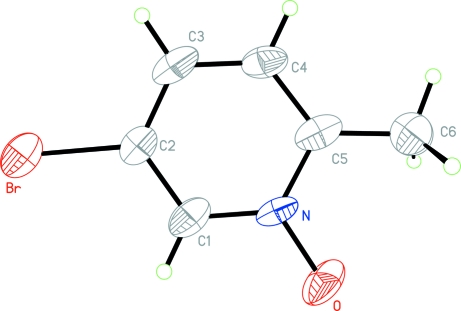
The molecular structure of the title molecule, with the atom-numbering scheme. Displacement ellipsoids are drawn at the 30% probability level.

**Fig. 2.**
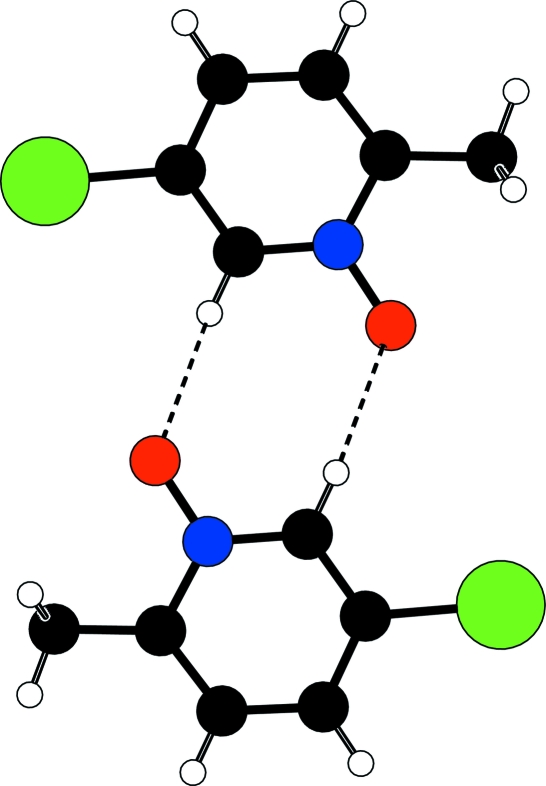
A partial packing diagram of (I). Hydrogen bonds are shown as dashed lines.

## supplementary crystallographic information

### Comment

Some derivatives of pyridine are important chemical materials. We report herein the crystal structure of the title compound, (I).

In the molecule of (I), (Fig. 1), ring A (N/C1-C5) is, of course, planar. Br atom is at a distance of -0.103 (3) Å to the plane of ring A, while atoms O and C6 lie in the ring plane.

In the crystal structure, intermolecular C-H···O hydrogen bonds (Table 1) link the molecules into centrosymmetric dimers (Fig. 2), in which they may be effective in the stabilization of the structure.

### Experimental

For the preparation of the title compound, 5-bromo-2-methylpyridine (80 g, 462 mmol) was suspended in glacial acetic acid (300 ml), aqueous hydrogen peroxide (35%) was added and the mixture was heated in a water-bath at 343-353 K. After 3 h a further hydrogen peroxide solution (35 ml) was added and the mixture was maintained an additional 9 h at the same temperature. The mixture was concentrated to about 100 ml, diluted with water (100 ml), and then again concentrated in vacuum as far as possible upon cooling to room temperature, a precipitate formed, which was collected by filtration, and then washed with cold ethanol (2 × 50 ml) to afford the ethyl ester as a white solid (yield; 83 g, 95%) (Ochiai, 1953).  Crystals of (I) suitable for X-ray analysis were obtained by slow evaporation of a methanol solution.

### Refinement

H atoms were positioned geometrically, with C-H = 0.93 and 0.96 Å for aromatic and methyl H, respectively, and constrained to ride on their parent atoms with U_iso_(H) = xU_eq_(C), where x = 1.5 for methyl H, and x = 1.2 for aromatic H atoms.

### Crystal data

**Table:**

C_6_H_6_BrNO	*F*_000_ = 368
*M**_r_* = 188.03	*D*_x_ = 1.899 Mg m^−^^3^
Monoclinic, *P*2_1_/*n*	Mo *K*α radiation λ = 0.71073 Å
Hall symbol: -P 2yn	Cell parameters from 25 reflections
*a* = 7.3060 (15) Å	θ = 10–13º
*b* = 11.351 (2) Å	µ = 6.16 mm^−^^1^
*c* = 8.4950 (17) Å	*T* = 294 (2) K
β = 111.01 (3)º	Block, colorless
*V* = 657.7 (3) Å^3^	0.10 × 0.05 × 0.05 mm
*Z* = 4	

### Data collection

**Table:**

Enraf–Nonius CAD-4 diffractometer	*R*_int_ = 0.036
Radiation source: fine-focus sealed tube	θ_max_ = 25.2º
Monochromator: graphite	θ_min_ = 3.1º
*T* = 294(2) K	*h* = −8→8
ω/2θ scans	*k* = 0→13
Absorption correction: ψ scan(North *et al.*, 1968)	*l* = 0→10
*T*_min_ = 0.578, *T*_max_ = 0.748	3 standard reflections
1275 measured reflections	every 120 min
1180 independent reflections	intensity decay: none
747 reflections with *I* > 2σ(*I*)	

### Refinement

**Table:**

Refinement on *F*^2^	Secondary atom site location: difference Fourier map
Least-squares matrix: full	Hydrogen site location: inferred from neighbouring sites
*R*[*F*^2^ > 2σ(*F*^2^)] = 0.063	H-atom parameters constrained
*wR*(*F*^2^) = 0.162	*w* = 1/[σ^2^(*F*_o_^2^) + (0.08*P*)^2^ + *P*] where *P* = (*F*_o_^2^ + 2*F*_c_^2^)/3
*S* = 1.05	(Δ/σ)_max_ < 0.001
1180 reflections	Δρ_max_ = 0.87 e Å^−^^3^
76 parameters	Δρ_min_ = −0.69 e Å^−^^3^
Primary atom site location: structure-invariant direct methods	Extinction correction: none

### Special details

**Table:**

Geometry. All e.s.d.'s (except the e.s.d. in the dihedral angle between two l.s. planes) are estimated using the full covariance matrix. The cell e.s.d.'s are taken into account individually in the estimation of e.s.d.'s in distances, angles and torsion angles; correlations between e.s.d.'s in cell parameters are only used when they are defined by crystal symmetry. An approximate (isotropic) treatment of cell e.s.d.'s is used for estimating e.s.d.'s involving l.s. planes.
Refinement. Refinement of F^2^ against ALL reflections. The weighted R-factor wR and goodness of fit S are based on F^2^, conventional R-factors R are based on F, with F set to zero for negative F^2^. The threshold expression of F^2^ > 2sigma(F^2^) is used only for calculating R-factors(gt) etc. and is not relevant to the choice of reflections for refinement. R-factors based on F^2^ are statistically about twice as large as those based on F, and R- factors based on ALL data will be even larger.

### Fractional atomic coordinates and isotropic or equivalent isotropic displacement parameters (Å^2^)

**Table:**

	*x*	*y*	*z*	*U*_iso_*/*U*_eq_	
Br	0.17025 (14)	0.74086 (7)	0.64566 (12)	0.0448 (4)	
N	0.6201 (9)	0.9804 (5)	0.7872 (8)	0.0290 (15)	
O	0.6767 (10)	1.0662 (5)	0.8994 (8)	0.0506 (18)	
C1	0.4547 (13)	0.9187 (7)	0.7722 (10)	0.038 (2)	
H1A	0.3842	0.9381	0.8405	0.046*	
C2	0.3901 (11)	0.8283 (6)	0.6579 (10)	0.0289 (18)	
C3	0.4939 (15)	0.7989 (8)	0.5576 (12)	0.045 (2)	
H3A	0.4523	0.7389	0.4781	0.053*	
C4	0.6635 (12)	0.8623 (7)	0.5795 (11)	0.038 (2)	
H4A	0.7354	0.8418	0.5127	0.045*	
C5	0.7341 (13)	0.9535 (7)	0.6929 (10)	0.036 (2)	
C6	0.9107 (13)	1.0232 (8)	0.7239 (12)	0.047	
H6A	0.9754	0.9981	0.6493	0.071*	
H6B	0.9971	1.0126	0.8387	0.071*	
H6C	0.8761	1.1050	0.7043	0.071*	

### Atomic displacement parameters (Å^2^)

**Table:**

	*U*^11^	*U*^22^	*U*^33^	*U*^12^	*U*^13^	*U*^23^
Br	0.0512 (6)	0.0387 (6)	0.0354 (6)	−0.0061 (5)	0.0045 (4)	0.0010 (5)
N	0.035 (4)	0.022 (3)	0.022 (4)	0.009 (3)	0.000 (3)	−0.006 (3)
O	0.072 (5)	0.042 (4)	0.032 (4)	−0.021 (3)	0.011 (4)	−0.016 (3)
C1	0.050 (6)	0.030 (4)	0.027 (5)	−0.004 (4)	0.004 (4)	−0.002 (4)
C2	0.032 (4)	0.020 (4)	0.025 (4)	0.006 (3)	−0.002 (4)	0.006 (3)
C3	0.050 (6)	0.035 (5)	0.036 (5)	0.003 (5)	0.001 (5)	−0.014 (4)
C4	0.038 (5)	0.037 (5)	0.034 (5)	0.013 (4)	0.009 (4)	−0.008 (4)
C5	0.051 (5)	0.025 (4)	0.021 (5)	0.011 (4)	0.000 (4)	0.003 (4)
C6	0.047	0.047	0.047	0.000	0.017	0.000

### Geometric parameters (Å, °)

**Table:**

Br—C2	1.859 (8)	C3—H3A	0.9300
N—O	1.322 (8)	C4—C5	1.382 (12)
N—C1	1.362 (10)	C4—H4A	0.9300
N—C5	1.381 (10)	C5—C6	1.455 (12)
C1—C2	1.375 (11)	C6—H6A	0.9600
C1—H1A	0.9300	C6—H6B	0.9600
C2—C3	1.369 (12)	C6—H6C	0.9600
C3—C4	1.387 (12)		
			
O—N—C1	118.9 (7)	C5—C4—C3	125.0 (8)
O—N—C5	118.9 (7)	C5—C4—H4A	117.5
C1—N—C5	122.1 (7)	C3—C4—H4A	117.5
N—C1—C2	121.2 (8)	N—C5—C4	114.7 (8)
N—C1—H1A	119.4	N—C5—C6	117.1 (7)
C2—C1—H1A	119.4	C4—C5—C6	128.2 (8)
C3—C2—C1	119.7 (8)	C5—C6—H6A	109.5
C3—C2—Br	119.7 (6)	C5—C6—H6B	109.5
C1—C2—Br	120.6 (6)	H6A—C6—H6B	109.5
C2—C3—C4	117.3 (8)	C5—C6—H6C	109.5
C2—C3—H3A	121.4	H6A—C6—H6C	109.5
C4—C3—H3A	121.4	H6B—C6—H6C	109.5
			
O—N—C1—C2	−179.7 (7)	O—N—C5—C4	179.7 (7)
C5—N—C1—C2	−1.9 (12)	C1—N—C5—C4	2.0 (11)
N—C1—C2—C3	0.3 (12)	O—N—C5—C6	−0.3 (11)
N—C1—C2—Br	177.3 (6)	C1—N—C5—C6	−178.1 (7)
C1—C2—C3—C4	1.0 (13)	C3—C4—C5—N	−0.5 (13)
Br—C2—C3—C4	−176.0 (6)	C3—C4—C5—C6	179.5 (9)
C2—C3—C4—C5	−0.9 (14)		

### Hydrogen-bond geometry (Å, °)

**Table:**

*D*—H···*A*	*D*—H	H···*A*	*D*···*A*	*D*—H···*A*
C1—H1A···O^i^	0.93	2.41	3.264 (11)	153

Symmetry codes: (i) −*x*+1, −*y*+2, −*z*+2.
